# Brain Correlates of Human Leukocyte Antigen (HLA) Protection in Gulf War Illness (GWI)

**DOI:** 10.1016/j.ebiom.2016.10.019

**Published:** 2016-10-14

**Authors:** Lisa M. James, Brian E. Engdahl, Arthur C. Leuthold, Apostolos P. Georgopoulos

**Affiliations:** a Brain Sciences Center, Department of Veterans Affairs Health Care System, Minneapolis, MN 55417, USA; b Center for Cognitive Sciences, University of Minnesota, Minneapolis, MN 55455, USA; c Department of Psychiatry, University of Minnesota Medical School, Minneapolis, MN 55455, USA; d Department of Neuroscience, University of Minnesota Medical School, Minneapolis, MN 55455, USA; e Department of Psychology, University of Minnesota, Minneapolis, MN 55455, USA; f Department of Neurology, University of Minnesota Medical School, Minneapolis, MN 55455, USA

**Keywords:** Gulf War Illness (GWI), Human Leukocyte Antigen, Magnetoencephalography, Veterans

## Abstract

**Background:**

We recently reported that six alleles from class II genes of the Human Leukocyte Antigen (HLA) confer protection from Gulf War Illness (GWI) (Georgopoulos et al., 2015). The most significant effect is exerted on Neurological-Cognitive-Mood (NCM), Pain, and Fatigue symptoms, such that higher number of copies of the protective alleles are associated with lower symptom severity. Here we tested the hypothesis that this effect is exerted by modulating the strength of neural synchronicity.

**Methods:**

Eighty-one Gulf War veterans (65 with GWI and 16 healthy controls) underwent a magnetoencephalography (MEG) scan to assess the strength of brain synchronicity by computing zero-lag crosscorrelations (and their Fisher *z* transforms) between prewhitened MEG time series. A high-resolution HLA genotyping determined the number of copies, *k*, of the 6 protective alleles above in each participant. We tested the hypothesis above by regressing NCM, Pain and Fatigue symptom severity against the interaction term, *k* × *z* (HLA-related effect), while including *z* (non-HLA-related effect), gender and age as covariates. The *k* × *z* and *z* terms assessed HLA- and non-HLA-related effects, respectively, of neural synchronicity on symptom severity. The distributions of these effects in sensor space were visualized using statistical heatmaps.

**Findings:**

We found significant, graded HLA- and non-HLA-related effects: (a) NCM > Pain > Fatigue for HLA-related effects, (b) NCM > Fatigue > Pain for non-HLA-related effects, and (c) HLA-related > non-HLA-related effects for all symptoms. These effects had widespread but distinct distributions in sensor space that allowed the orderly separation of the 6 terms (3 symptom domains × 2 HLA factors) in a multidimensional plot, where one dimension separated the symptoms and the other the HLA relation.

**Interpretation:**

These findings demonstrate the presence of substantial, widespread, distinct and orderly HLA- and non-HLA-related neural influences on NCM, Pain and Fatigue symptom severity in GWI.

**Funding:**

U.S. Department of Veterans Affairs and University of Minnesota.

## Highlights

• Genetic modulation of neural synchronicity is investigated in Gulf War Illness (GWI).
• GWI symptom severity is highly related to specific Human Leukocyte Antigen (HLA) alleles.
• The distribution of HLA-related effects on brain regions differed across GWI symptoms.
• Non-HLA genetic effects on brain functioning were also observed.

Gulf War Illness (GWI) is a chronic and debilitating condition that has affected a large number of veterans who served during the 1990–91 Persian Gulf War. We have recently reported evidence of brain anomalies in GWI and identified a genetic immune protection the lack of which confers susceptibility, and, hence, risk for developing GWI symptoms. In this study, we evaluated the interaction of immunity (Human Leukocyte Antigen, HLA) genes and brain functioning in veterans with and without GWI using magnetoencephalography. The findings indicate genetic modulation of brain functioning on GWI symptoms, particularly with genes involving the HLA immune system. These findings document symptom-related interactions between HLA genes and brain function, thus forging a link between HLA genes, brain function and GWI.

## 1 Introduction

Gulf War Illness (GWI) is a disorder that affects multiple systems and is manifested by various combinations and severities of at least 6 different kinds of symptoms, including Neurological-Cognitive-Mood (NCM), pain, fatigue, skin rashes, gastrointestinal, and respiratory symptoms (Institute of Medicine National Research Council, 2000, Institute of Medicine National Research Council, 2006, Institute of Medicine National Research Council, 2010). Based on the presence of such symptoms, criteria have been developed to establish the diagnosis of GWI (Fukuda et al., 1998, Steele, 2000). The cause (or causes) of GWI are unknown, as is the pathophysiology of the disorder. Diverse lines of evidence have implicated three major factors as triggers, including (i) various vaccinations (Georgopoulos et al., 2015, Israeli, 2012, Toubi, 2012), (ii) various chemical exposures (Institute of Medicine National Research Council, 2000, Steele et al., 2015) and (iii) various kinds of stress, and, typically, combinations thereof, since all GW veterans were vaccinated, exposed to low doses of nerve gas, and subjected to strenuous basic training. Beyond specific effects of each one of these factors (White et al., 2016), a common denominator shared by all is their effects on the immune system. These effects are diverse, depending on the factor. Vaccinations obviously target the immune system by inducing immune responses to the pathogens, but also to the adjuvants, contained in the vaccines (Israeli, 2012, Toubi, 2012); chemical exposure to subclinical levels of sarin have been shown to depress immune function (Henderson et al., 2001); and stress has since long been identified as affecting immune function (see reviews by O'Leary, 1990, Webster Marketona and Glasera, 2008). Recent studies have documented immune dysfunction in GWI (Whistler et al., 2009), and even identified an immunosuppressant (methotrexate) as an optimal possible treatment for GWI (Craddock et al., 2015).

Recently, we focused our research on a different aspect of immune function, at the intersection of immunity and genetics, namely on the Human Leukocyte Antigen (HLA) (Georgopoulos et al., 2015). HLA genes are located in the Major Histocompatibility Complex (MHC) of chromosome 6 and play a central role in immune recognition (Meuer et al., 1982). HLA genes mediate all specific immune responses to intracellular and extracellular substances (Blum et al., 2013). Given the variety of factors above affecting immunity to which even nondeployed GW veterans were exposed, we reasoned that an investigation of the HLA genetic makeup in GW veterans, including healthy controls and veterans suffering from GWI, might reveal a clue on the susceptibility of GW veterans to develop GWI following immune challenges, as those above. Indeed, we identified 6 Class II HLA alleles which, when present, conferred protection from GWI. Presence or absence of those alleles classified correctly healthy and GWI veterans, respectively and, depending of the number of copies of alleles present, conferred graded alleviation in GWI symptom severity (Georgopoulos et al., 2015). Those alleles were: DRB1*01:01, DRB1*08:11, DRB1*13:02, DQB1*02:02, DPB*01:01, and DPB1*06:01. Since an individual has 2 copies of Class II HLA genes, and since HLA genes are highly polymorphic, the number of copies, collectively, of the 6 protective alleles that an individual can have is zero, one or two. We found that the overall GWI symptom severity decreased linearly and significantly as the number of copies of the 6 protective alleles increased. Of the various GWI symptom domains, HLA protection affected mostly the symptom severity of NCM, Pain and Fatigue. Since those symptoms are mediated through the brain, we sought to investigate the brain mechanisms involved in this protection. More specifically, we tested the hypothesis that this protection is exerted by modulating neural communication patterns, as assessed by brain's synchronous neural interactions, namely zero-lag, pairwise crosscorrelations calculated from prewhitened, 60-s resting-state magnetoencephalographic (MEG) recordings (Georgopoulos et al., 2007). These correlations have proved powerful in successfully discriminating various brain diseases (Georgopoulos et al., 2007), including posttraumatic stress disorder (PTSD) (Georgopoulos et al., 2010, Engdahl et al., 2010), and in identifying neural correlates of resilience to trauma (James et al., 2013), of posttraumatic growth (Anders et al., 2015), and of measures of psychopathology (James et al., 2015). More importantly, in a recent study of MEG patterns in GWI (Engdahl et al., 2016) we found synchronous crosscorrelations differed significantly between controls and GWI patients and could classify successfully (> 93% correctly) control and GWI participants. These findings provided the framework for this study, which investigated more specifically the relations between these correlations and specific GWI symptomatology.

## 2 Materials and Methods

### 2.1 Study Participants

We studied a total of 81 veterans, 16 controls (15 men, 1 woman; age range 43–71 y; 54.9 ± 10.2 y, mean ± SD) and 65 GWI (63 men, 2 women; age range 39–76 y; 50.8 ± 7.9 y). (This is the same group studied in Georgopoulos et al., 2015, less one GWI participant for whom we did not have MEG scan.) All participants had deployed during the Gulf War and were free of autoimmune disease, including multiple sclerosis, lupus, rheumatoid arthritis, psoriasis, Sjögren's syndrome, or Graves disease. Assignment to control or GWI group was based on established criteria, as described in Georgopoulos et al. (2015); specifically, a participant was assigned to the GWI group when meeting either the Centers for Disease Control and Prevention (CDC) criteria (Fukuda et al., 1998) or the Kansas GWI case definition (Steele, 2000). For that purpose, participants completed a symptom presence/severity questionnaire developed for use in Kansas Gulf War veterans (Steele, 2000) that evaluates a range of symptoms associated with GWI and permits determination of case status according to either the CDC criteria (Fukuda et al., 1998) or the Kansas GWI case definition (Steele, 2000). The questionnaire asks participants to indicate if they have had a persistent problem over the last 6 months with various symptoms from the following six domains: fatigue, pain, neurological-cognitive-mood, skin, respiratory, and gastrointestinal. For each symptom rated as present, participants are asked to rate the severity of the symptom as absent, mild, moderate, or severe (scored as 0, 1, 2, or 3, respectively), and to indicate whether the symptom first became problematic before, during or after deployment to the Gulf. Only symptoms that began during or after Gulf War service are counted toward diagnosis. There were six symptom domains: NCM, Pain, Fatigue, Skin, Gastrointestinal, and Respiratory (Steele, 2000). Individual symptom severity was reported in a scale from 0 to 3, as described above. For each participant, an average score per domain was calculated. In this study, only the scores for NCM, Pain and Fatigue were used.

### 2.2 HLA Genotyping

DNA isolation was carried out from 3 ml of whole blood drawn in EDTA tubes, using a commercially available kit (ArchivePure cat. 2300730) from 5Prime (distributed by Fisher Scientific or VWR) with an expected yield of 50–150 μg of DNA. The purified DNA samples were sent to Histogenetics (http://www.histogenetics.com/) for high-resolution HLA Sequence-based Typing (SBT; details are given in https://bioinformatics.bethematchclinical.org/HLA-Resources/HLA-Typing/High-Resolution-Typing-Procedures/ and https://bioinformatics.bethematchclinical.org/WorkArea/DownloadAsset.aspx?id=6482). Their sequencing DNA templates are produced by locus- and group-specific amplifications that include exon 2 and 3 for Class I (A, B, C) and exon 2 for Class II (DRB1, DRB3/4/5, DQB1, and DPB1) and reported as Antigen Recognition Site (ARS) alleles as per ASHI recommendation (Cano et al., 2007).

### 2.3 Data Acquisition

As described previously (Georgopoulos et al., 2007, Georgopoulos et al., 2010), subjects lay supine within the electromagnetically shielded chamber and fixated their eyes on a spot ~ 65 cm in front of them, for 60 s. MEG data were acquired using a 248-channel axial gradiometer system (Magnes 3600WH, 4-D Neuroimaging, San Diego, CA), band-filtered between 0.1 and 400 Hz, and sampled at 1017.25 Hz. Data with artifacts (e.g. from non-removable metal or excessive subject motion) were eliminated from further analysis.

### 2.4 Data Analysis

#### 2.4.1 General

Standard statistical methods were used to analyze data (analysis of covariance [ANCOVA], correlation, etc.) (Snedecor and Cochran, 1980) using, as needed, the IBM-SPSS statistical package (version 24), the Matlab package (version R2015b), time series analysis programs written in Python (Mahan et al., 2015), and ad hoc Fortran computer programs employing the International Mathematics and Statistics Library (IMSL; Rogue Wave Software, Louisville, CO, USA) statistical and mathematical libraries.

#### 2.4.2 MEG Data

Single trial MEG data from all sensors underwent prewhitening (Box and Jenkins, 1976, Priestley, 1971) using a (50,1,3) ARIMA model (Mahan et al., 2015) to obtain practically white noise innovations (i.e. residuals). As we have argued previously (Engdahl et al., 2010), the high-frequency sampling of the MEG signal at a resting state (i.e. in the absence of time-locked strong signals aligned to a specific event), and its millisecond-by-millisecond differencing suggest that it originates from widely distributed, small intensity cortical generators reflecting integrated synaptic activity in small neuronal populations. For each brain scan, all possible pairwise zero-lag crosscorrelations *r*_*ij*_ between the innovations of *i* and *j* sensors (N = 30,628, given 248 sensors) were computed. Since, in this analysis, we were interested in the strength of neural synchronicity, irrespective of its sign, we took the absolute value *r*′_*ij*_ of *r*_*ij*_ and computed its Fisher (1958) *z*-transform to normalize its distribution:

(1) $$z_{\mathrm{ij}}=\text{atanh}\left({r\prime }_{\mathrm{ij}}\right)$$

#### 2.4.3 Testing for HLA-neural Interactions

**Fig. 1:**
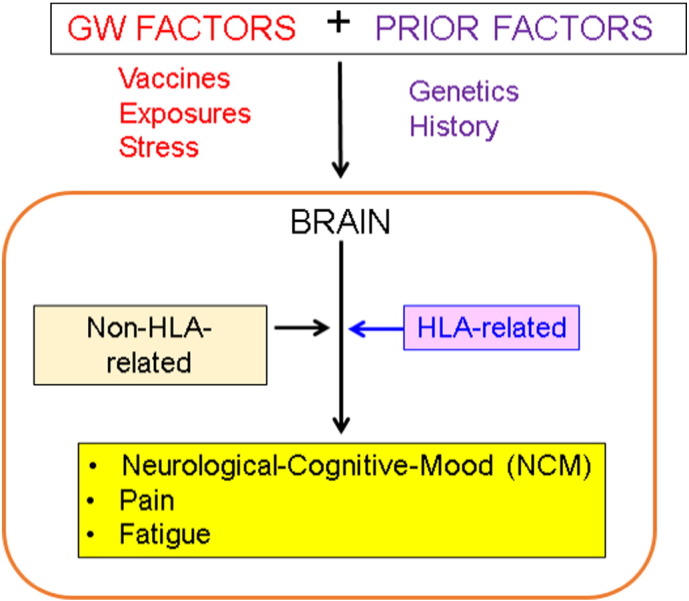
Scheme of working hypothesis regarding HLA-related and non-HLA-related effects on GWI NCM, Pain and Fatigue symptom severity.

Our working hypothesis on how symptom severity may be the outcome of HLA-related and non-HLA-related effects on brain function is illustrated in Fig. 1. We tested this hypothesis by performing three multiple linear regression where the dependent variable was NCM, Pain, or Fatigue symptom severity and the independent variables included a HLA-related variable (coded as an multiplicative interaction term, *kz*_*ij*_, where *k* is the number of copies of the 6 HLA protective alleles), a non-HLA-related variable (*z*_*ij*_), and age and gender as covariates:

(2) $$\text{Symptom severity}=a+b\left(kz_{\mathrm{ij}}\right)+cz_{\mathrm{ij}}+d\left(\mathrm{Age}\right)+e\left(\text{Gender}\right)+\hat{\varepsilon }$$

where *a* is a constant, *b–e* are partial regression coefficients, and $\hat{\varepsilon }$ is an error term. This analysis provided a general assessment of our hypothesis. A more detailed analysis was also performed using the same regression model above but applied separately to individual sensor pairs (N = 30,628 pairs from 248 sensors). This provided information concerning the distribution of HLA-related and non-HLA-related effects in the brain, as visualized in statistical heatmaps, constructed as follows. For each *i* MEG sensor, there are 247*z*_*ij*_, corresponding to the remaining 247 *j* sensors. Therefore, 247 multiple regressions were performed (Eq. (2)) for each sensor. This analysis gives a *t* value for each partial regression coefficient in Eq. (2) (i.e. the ratio of the coefficient over its standard error). The absolute *t* value, |* t* |, is essentially a signal-to-noise ratio that indicates the degree of confidence in the effect; corresponding probability values are uncorrected for multiple comparisons, since they are not used for specific hypothesis testing. In a heatmap, the color at a sensor location was varied according the maximum |* t* | value of the partial regression coefficient for a specific effect (i.e., *b* and *c* coefficients in Eq. (2) for a HLA- and non-HLA-related effects, respectively). Finally, the |* t* | values were used in a multidimensional scaling (MDS) analysis aimed to visualize in the same plot the symptom domain (NCM, Pain, Fatigue) and the HLA-neural dependence (HLA-related, non-HLA-related), as follows. Each heatmap consisted of 248 |* t* | values, one per MEG sensor. A 6 × 6 proximity (similarity) matrix was constructed consisting of all 15 pairwise coefficients of determination (*r*^2^) between the 6 Symptom × HLA-effect combinations. This matrix was then fed as input to the IBM-SPSS procedure PROSCAL using a non-metric (ordinal) MDS model which yielded a 2-D plot of the 6 combinations and associated goodness of fit statistics.

## 3 Results

The two groups in this study (control and GWI) differed substantially in their composition regarding the counts of 6 HLA protective alleles (Table 1): controls had higher counts (including 3 participants with 2 alleles), whereas GWI participants had lower counts, without a single participant with 2 alleles. This differential count distribution was highly statistically significant (Chi-square test, *χ*_[2]_^2^ = 21.9 , *P* = 0.000018 ).

### 3.1 General Effects

**Fig. 2:**
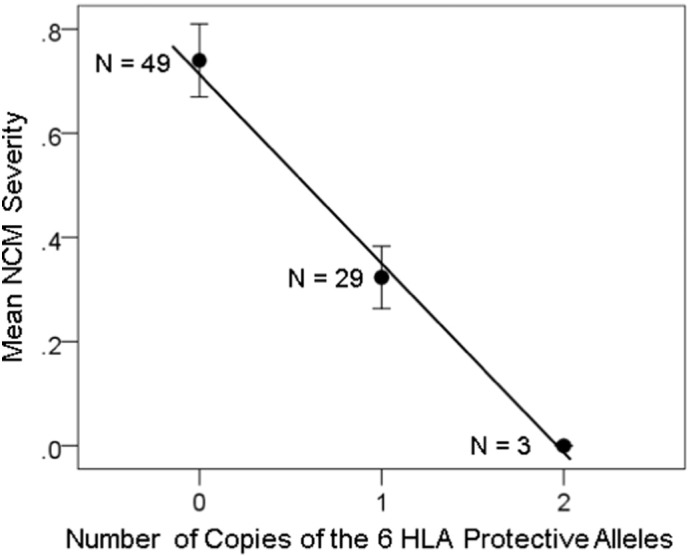
NCM symptom severity (± SEM) is plotted against the number of copies of the 6 protective HLA alleles. N refers to the number of participants. *R*^2^ = 0.221 , *P* = 0.00065.

**Fig. 3:**
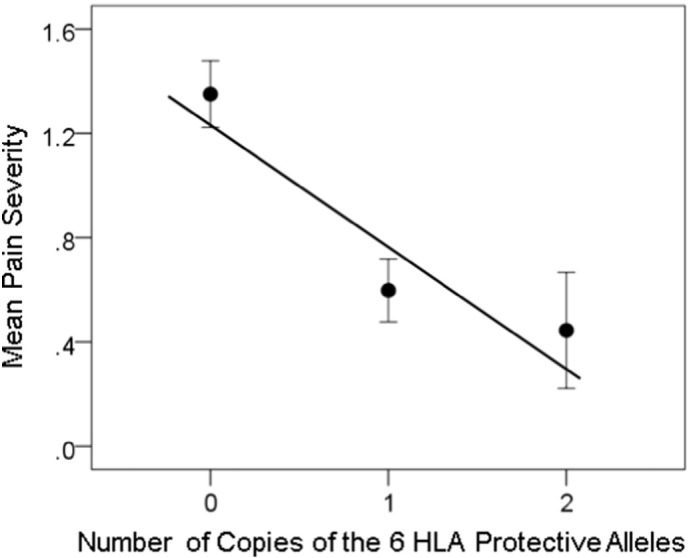
Pain severity (± SEM) is plotted against the number of copies of the 6 protective HLA alleles. N refers to the number of participants. *R*^2^ = 0.174 , *P* = 0.0001.

**Fig. 4:**
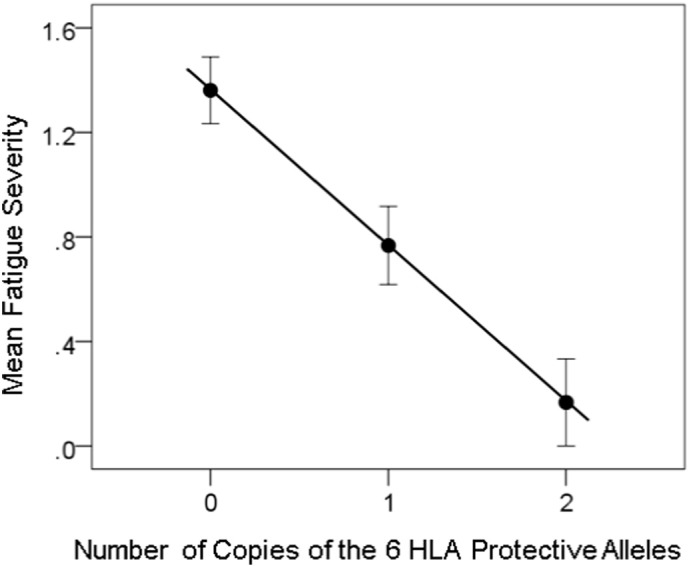
Fatigue severity (± SEM) is plotted against the number of copies of the 6 protective HLA alleles. N refers to the number of participants. *R*^2^ = 0.142 , *P* = 0.001.

The dependence of NCM, Pain and Fatigue severity on the number of copies of the 6 protective HLA alleles is shown in Fig. 2, Fig. 3, Fig. 4. The similarity of the fitted lines stems from the fact that symptom severity was significantly correlated (*P* < 0.001) among the three domains (*r* = 0.604 , 0.698 , 0.567 for NCM vs. Pain, NCM vs. Fatigue, and Pain vs. Fatigue, respectively), a typical feature of GWI. With respect to the brain, there was an overall strong and highly significant effect of the HLA-related interaction (*kz*_*ij*_ term in Eq. (2)) on symptom severity (standardized partial regression coefficient |* β* | = 0.274 , 0.232 , and 0.200 for NCM, Pain and Fatigue, respectively, *P* < 0.001 for all three coefficients). There was also a weaker effect of the non-HLA-related term *z*_*ij*_: (|* β* | = 0.156 , 0.136 , and 0.109 for NCM, Pain, and Fatigue, respectively, P < 0.001 for all three coefficients). There was no significant collinearity between *kz*_*ij*_ and *z*_*ij*_ (tolerance for both terms = 0.7 in all regression analyses).

### 3.2 Regional Distribution of Effects

**Fig. 5:**
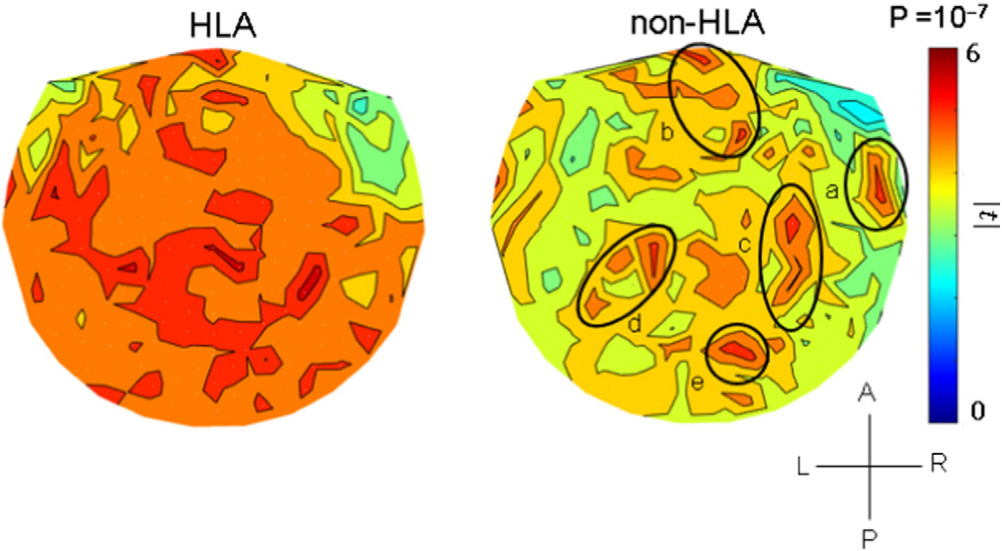
Heatmaps of HLA-related and non-HLA-related effects on NCM severity. Color scale indicates maximum absolute value of the t-statistic (or its corresponding uncorrected probability value) for a given sensor location. (See text for details.) Approximate areas of large effects are demarcated by circles. a, anterior temporal lobe; b, medial frontal cortex; c–e, posterior parietal and occipital cortex.

**Fig. 6:**
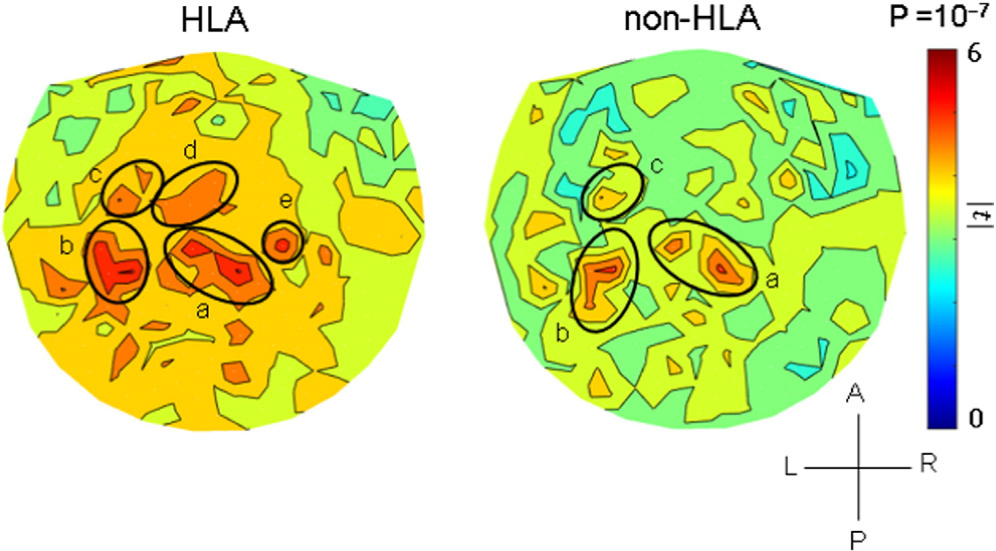
Heatmaps of HLA-related and non-HLA-related effects on Pain severity. Color scale indicates maximum absolute value of the t-statistic for a given sensor location. (See text for details.) All areas in both plots are in parietal cortex.

**Fig. 7:**
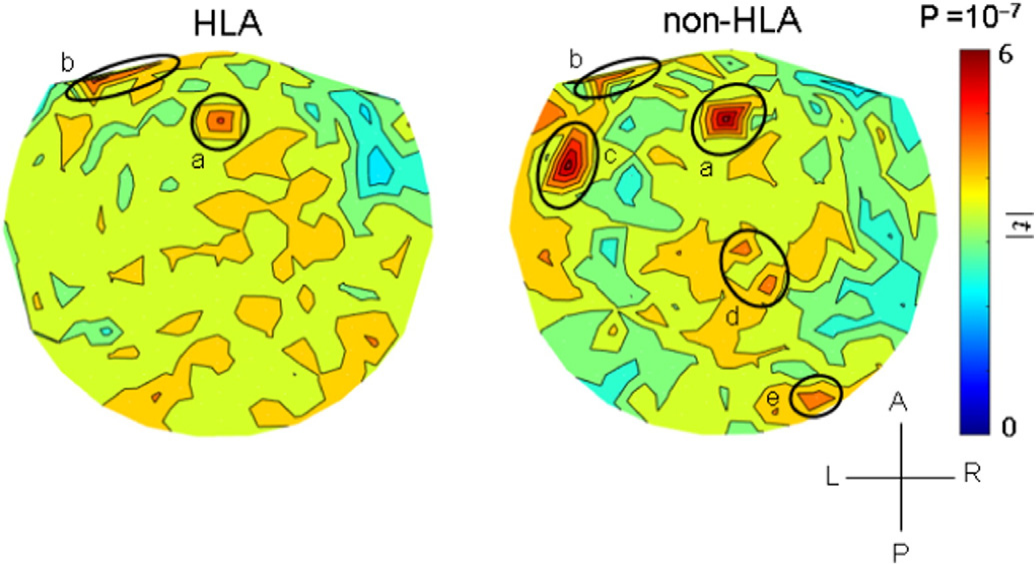
Heatmaps of HLA-related and non-HLA-related effects on Fatigue severity. Color scale indicates maximum absolute value of the t-statistic for a given sensor location. (See text for details.) a, medial frontal cortex; b, prefrontal cortex; c, temporal cortex; d, posterior parietal cortex; e, cerebellum.

**Fig. 8:**
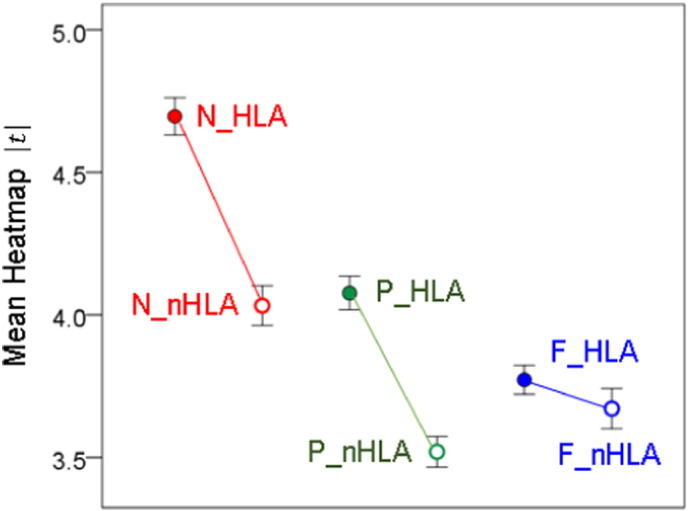
Mean (± 95% confidence intervals) heatmap |* t* | value, from maps in Fig. 5, Fig. 6, Fig. 7.

The results of the analyses per sensor are illustrated in the heatmaps of Fig. 5, Fig. 6, Fig. 7 and summarized in Fig. 8. It can be seen that, in general, HLA-related effects were stronger than non-HLA effects (left vs. right panels in Fig. 5, Fig. 6, Fig. 7, and filled vs. open circles in Fig. 8). Interestingly, all HLA-related effects spared the right anterior temporal lobe. More specifically, the following can be seen. (a) For NCM (Fig. 5), the HLA-effects were strong and widespread, whereas the non-HLA-related effects were mostly focused and centered on the right anterior temporal lobe, medial prefrontal cortex, posterior parietal and occipital cortex. (b) For Pain (Fig. 6), the HLA-related effects are stronger than the non-HLA-related effects but are similarly distributed in central parietal areas. Finally, for Fatigue (Fig. 7), although HLA-related effects are stronger on the average (Fig. 8), the non-HLA related effects are stronger in specific areas. Both HLA- and non-HLA-related effects involve medial and left prefrontal areas, non-HLA-related effects involve, in addition, left temporal cortex, right posterior parietal cortex and right cerebellum.

### 3.3 Multidimensional Scaling Analysis

**Fig. 9:**
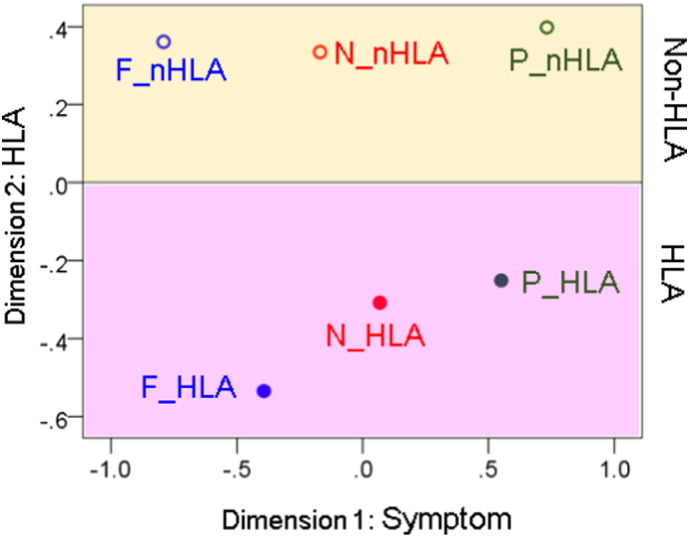
MDS object plot of the six Symptom × HLA combinations. (See text for detail.)

The results of the MDS analysis are shown in Fig. 9. The proximity (similarity) matrix that was used as the input to this analysis is given in Table 2. It consists of the correlation coefficients between the 248 t-values of each heatmap (Fig. 5, Fig. 6, Fig. 7), It can be seen that MDS identified successfully the 2 dimensions of the data, namely the symptom domain (Dimension 1) and the HLA relation (Dimension 2). The fit was excellent (normalized raw stress = 0.0083, Dispersion Accounted For = 0.99).

## 4 Discussion

Here we evaluated the interactive effects of six Class II HLA alleles and neural synchrony on NCM, Pain and Fatigue symptom severity in Gulf War veterans. Our results demonstrated a highly significant effect of the HLA × neural synchrony interaction, particularly on NCM scores which revealed widespread effects involving much of the cortex and the cerebellum. A limitation of the study concerns the relatively small sample size (N = 81 participants); a larger study is needed to further validate these findings.

### 4.1 NCM, Pain and Fatigue Symptom Severity is Associated with HLA-related Modulation of Neural Synchrony

We previously demonstrated protective effects of certain Class II HLA alleles on several GWI symptom domains, the most prominent of which included NCM, Pain and Fatigue (Georgopoulos et al., 2015). Specifically, symptom severity decreased with the number of copies (zero, one, or two) of six HLA alleles that successfully discriminated GWI from control participants. The present study demonstrated that protection was conferred via genetic modulation of neural communication. Synchronous neural activity is central to several cognitive functions including attention, memory, and sensory-motor integration. Cognitively healthy individuals display remarkably similar patterns of neural synchronicity (Langheim, 2006), whereas alterations in neural synchrony have been associated with several neuropsychiatric disorders (Engdahl et al., 2010, Georgopoulos et al., 2007, Georgopoulos et al., 2010, James et al., 2013, 2015; Uhlhaas and Singer, 2006). We have recently demonstrated that GWI is also associated with aberrant synchronicity affecting cortical areas and the cerebellum (Engdahl et al., 2016). Our working hypothesis (Fig. 4) is that, when exposed to certain triggers (e.g., vaccines, chemical exposures, and stress), genetically (HLA) vulnerable veterans exhibit widespread synchronicity anomalies that contribute to the diverse problems included under the NCM, Pain and Fatigue domains, and, conversely, the presence of protective HLA alleles would prevent these anomalies. Our analyses teased out such HLA-related effects from non-HLA-related effects. These two kinds of effects differed according to the symptom domain. The symptom of Pain (Fig. 6) was the simplest one: the patterns of regional distribution of the effects were very similar (areas a, b, c in left and right panels of Fig. 6) but stronger and a bit more widespread (areas d and e in the left panel of Fig. 6) in the HLA-related than in the non-HLA-related case. These effects encompassed parietal areas (tentatively, first [area c in Fig. 6] and second somatosensory cortex [area b]), known to be involved in pain (Alonso et al., 2010, Mazzola et al., 2012). The preponderance of left hemispheric involvement in this study (Fig. 6) is difficult to explain and remains to be further investigated. It is possible that this may reflect an engagement of language/verbalization in coping with chronic pain.

Concerning Fatigue (Fig. 7), the interplay between HLA- and non-HLA-related effects was reversed, in that effects in specific common areas involved (medial frontal and left prefrontal, areas labeled a and b, respectively, in the left and right panels of Fig. 7) were stronger in the non-HLA-related than the HLA-related case. Moreover, there were three additional foci in the non-HLA-related effects, most notably in the left temporal cortex. However, there were, overall, more widespread effects in the HLA-related case, leading to a stronger average effect (Fig. 8).

Finally, the NCM case (Fig. 5) demonstrates a strong and pervasive HLA-related effect, in contrast to weaker, localized non-HLA-effects involving specific areas, most notably the anterior right temporal lobe (labeled a in the right panel of Fig. 5), medial prefrontal cortex (area b), posterior parietal areas bilaterally (areas c, d), and right occipital cortex (area e). This distribution is very similar to that observed in uncomplicated PTSD (see Fig. 2 in Engdahl et al., 2010) and reflects deficits in decision making, attention and memory, to name but a few functions subserved by the areas above. Specifically, the involvement of areas a, c, d and e in the right panel of Fig. 5 are clearly overlapping with those in Fig. 2 of Engdahl et al. (2010). It is noteworthy that all non-HLA-related effects (right panel of Fig. 5) were present and stronger in the HLA-related heat map (left panel of Fig. 5) except for the effect involving the right anterior temporal lobe which was practically absent in the HLA-related case.

### 4.2 Multidimensional Scaling

The heatmaps of Fig. 5, Fig. 6, Fig. 7 share similarities and differences reflecting HLA- and non-HLA-related associations of brain synchronicity to symptom severity. However, they do not, at first glance, convey, collectively, any order or arrangement regarding shared aspects of their origin, namely the symptom domain or HLA relation to which they belong. We sought to derive such an arrangement by applying MDS, a venerable method for uncovering basic dimensions in a multidimensional dataset (Shepard, 1980, Shepard, 1988). The MDS is a well-established dimension-reduction method (Borg and Groenen, 2010) that has proved valuable in psychological (Shepard, 1980, Shepard, 1988, Whang et al., 1999), psychiatric (Stephane et al., 2003), and neuroscientific applications (Leuthold et al., 2013, Tagaris et al., 1998, Tzagarakis et al., 2009, Young, 1992, Young and Yamane, 1992, Young et al., 1995). It typically reveals underlying dimensions along which variables are ordered, dimensions that may be hidden when embedded in a multidimensional space. Our MDS analysis of the associations among the six heatmaps (Fig. 5, Fig. 6, Fig. 7) revealed a striking arrangement (Fig. 9) which cleanly ordered the symptom domain and HLA relation in two dimensions with high goodness of fit. This result further validates the statistical analyses that led to the construction of the heat maps and underscores the internal consistency of the derived associations.

### 4.3 HLA-related Brain Mechanisms in GWI

The nature of the mechanisms by which HLA effects are exerted are unknown. Given the role of HLA in immune function, and the fact that our 6 protective alleles are all from HLA class II genes (Georgopoulos et al., 2015), it is reasonable to suppose that the HLA-conferred protection in GWI is mediated through specific, adaptive immunity mechanisms, manifested in successful elimination of pathogens. We assume that our alleles were instrumental in that process, since their absence is associated with the presence of GWI and high severity of GWI symptoms. It is reasonable, then, to suppose that this is due to pathogens that could not be eliminated in the absence of those alleles. There are two obvious mechanisms for this effect. First, if pathogens are not eliminated, they are allowed to persist in the body, even in a relatively inactivated form, and produce some harm. And second, their persistence may trigger the production of autoantibodies, if the pathogens happen to share common antigenic structure with body proteins, and this may lead to autoimmunity. The most obvious potential pathogens for Gulf War veterans include the vaccines administered (multiply in some veterans, see Institute of Medicine, 2000) and their adjuvants (Asa et al., 2000). The potential adverse role of the various vaccinations is described in detail in the report by the Institute of Medicine (2000), and specific mention is made of HLA in that context. At that time, research on HLA's role in disease was focused on discovering HLA genes associated with it, an ongoing, perennial focus in the effort to understand disease pathogenesis till now. However, the fundamental and overall role of HLA, and associated T lymphocyte mechanisms, is basically protective, namely to eliminate offending pathogens. If that is lacking genetically, as, for example in bare lymphocyte syndrome (DeSandro et al., 1999, Reith and Mach, 2001) or compromised (e.g. by administration of immunosuppressive medications, infection by human immunodeficiency virus, etc.), increased risk to infections ensues. In GWI, an increased risk for various infections has been reported (Institute of Medicine National Research Council, 2010, Nicolson et al., 2000, Nicolson et al., 2002), suggesting a reduced ability to deal effectively with infections, probably due to immune dysfunction. Now, there is a link between chronic infections and the development of autoimmune disorders (Sherbet, 2009). In addition, a possible, direct connection of GWI to autoimmunity has been suggested (Asa et al., 2000, Israeli, 2012, Georgopoulos et al., 2015) and supported by the emergence of methotrexate, an immunosuppressive drug, as a possible treatment for GWI (Craddock et al., 2015).

This connection of GWI to autoimmunity is probably the best explanation of the HLA-related neural effects found in this study. Specifically, we hypothesize that, in the absence of HLA protection, brain autoimmunity develops in GWI, i.e. antibodies against antigens in the brain, resulting in cellular abnormalities, abnormal neural communication, and symptomatology – all graded according to the level of protection present (i.e. 0, 1 or 2 protective allele copies). At face value, neural communication is commonly assessed by calculating the correlation coefficient, and it is the modulation of the correlation between MEG time series that we have identified here as a significant factor associated with GWI symptomatology. Now, the correlation coefficient between two variables is calculated as the ratio of their covariance over the product of the square root of their variances. Therefore, a change in correlation can stem from a change in covariance, the variances, or both. These possibilities have different implications; for example, a change in covariance would point to an alteration in network functional interactions, whereas a change in variance would indicate an alteration in local neural processing. Both mechanisms would make the correlation stronger but for different reasons. Although the specific cellular/biochemical mechanisms for such changes in correlation are unknown, they are likely to share similar mechanisms that underlie brain dysfunction in other autoimmune or inflammatory brain disorders (Selmi et al., 2016), including neuropsychiatric lupus (Belmont et al., 1996, Menon et al., 1999, Omdal et al., 2005), rheumatoid arthritis (Shin et al., 2012), Sjögren's syndrome (Tezcan et al., 2016), and psoriasis (Gisondi et al., 2014). A special case is autoimmune encephalitis where the investigation of cellular autoimmune mechanisms has progressed appreciably (Armangue et al., 2015, Boronat et al., 2013, Clemente-Casares et al., 2016, Dalmau and Rosenfeld, 2008, Dalmau and Rosenfeld, 2014, Linnoila et al., 2014).

### 4.4 Non-HLA-related Brain Mechanism in GWI

Our analyses distinguished HLA-related from non-HLA-related associations between GWI NCM, Pain and Fatigue symptom severity and neural synchronicity. The non-HLA-related effects for NCM and Pain are interpretable based on prior knowledge concerning the neural mechanisms underlying such symptoms. For example, the pattern in the non-HLA-related heatmap for NCM (right panel of Fig. 5) is very similar to that we reported for uncomplicated PTSD (Fig. 2 in Engdahl et al., 2010). This pattern is in clear contrast to the HLA-related effects (left panel in Fig. 5) which are stronger and much more widespread encompassing many areas but, conspicuously, sparing the right anterior temporal lobe, a strong focus of the non-HLA-related effect. This suggests that non-HLA-related effects reflect other factors, including possibly other genes and environmental factors, the nature of which remains to be determined. For instance, Steele et al. (2015) found elevated risk for GWI in veterans who reported warzone use of pyridostigmine bromide, an acetylcholinesterase inhibitor, and had a less common variant of the butyrylcholinesterase enzyme relative to veterans with other genotypes. The influence of other genes in promoting protection or susceptibility to GWI symptoms remains to be determined.

Concerning Pain (Fig. 6), non-HLA-related effects were as expected from the literature, namely encompassing parietal areas. This pattern was enhanced and extended in the HLA-related case, which thus added further sensitization but did not alter the basic pattern. In contrast, in the Fatigue case (Fig. 7), the distinct pattern observed for non-HLA-related effects (right panel in Fig. 7) was blurred in the HLA-related heatmap (left panel in Fig. 7) and substituted by widespread, relatively homogeneous effects.

## 5 Concluding Remarks

These findings underscore the diversity of brain mechanisms underlying GWI symptom severity and the complex interplay of HLA-related and non-HLA-related factors. Of course, the overall symptom severity reflects both of those factors but the separation in brain patterns we have documented opens the possibility of tailoring targeted therapeutic interventions at multiple, different levels, namely traditional approaches aimed to alleviate symptoms but also interventions targeting immune dysfunction aimed to correct cellular/biochemical abnormalities giving rise to the symptoms.

## Financial Disclosures

The authors do not report any financial disclosures.

## Author Contributions

Contributed to general data collection and clinical evaluation: LMJ, BEE. Contributed to MEG data collection: ACL. Contributed to study design: APG, LMJ BEE. Contributed to data analysis: APG, LMJ. Wrote the paper: APG, LMJ. Contributed to editing the paper: All.

## Role of the Funding Source

Partial funding for this study was provided by a service directed grant from the U.S. Department of Veterans Affairs and the University of Minnesota (Brain and Genomics Fund and the American Legion Brain Sciences Chair). The sponsors had no role in the current study design, analysis or interpretation, or in the writing of this paper. The contents do not represent the views of the U.S. Department of Veterans Affairs or the United States Government.

## Figures and Tables

**Table 1:** Counts of the 6 HLA protective alleles for the control and GWI groups.

HLA protective allele count	Control	GWI	Total
0	3	46	49
1	10	19	29
2	3	0	3
Total	16	65	81

**Table 2:** Correlation matrix between the six heatmaps of Fig. 5, Fig. 6, Fig. 7.

	HLA-NCM	HLA-Pain	HLA-Fatigue	nonHLA-NCM	nonHLA-Pain	nonHLA-Fatigue
HLA-NCM	1.000					
HLA-Pain	0.587	1.000				
HLA_Fatigue	0.613	0.357	1.000			
nonHLA-NCM	0.511	0.313	0.311	1.000		
nonHLA-Pain	0.291	0.447	0.157	0.281	1.000	
nonHLA-Fatigue	0.243	0.061	0.352	0.380	0.169	1.000
